# 

**Published:** 1901-02-09

**Authors:** 


### NOTICE TO CORRESPONDENTS.

All MSS., Letters, Books for Review, and other matters intended for the Editor should be addressed The Editor, "The Hospital," The Hospital Building, 28 & 29 Southampton Street, Strand, W.C.

The Editor cannot undertake to return rejected MS., even when accompanied by stamped directed envelope.

**Notice to Advertisers and Subscribers.**

All Advertisements, Orders for Copies of The Hospital, and Business Communications should be addressed to **THE MANAGER (Not to the Editor)**, The Hospital, 28 & 29 Southampton Street Strand, London, W.C.

The Cost of Subscription, post free, is as follows:—Per week, 2½d.; per quarter, 2s. 8d.; per half-year, 5s. 3d.; per annum, 10s. 6d. Foreign:—Per annum, 15s. 2d., payable, in all cases, in advance.

**NOTICE.—Vol. XXVIII. of "The Hospital" (April to September, 1900), in handsome cloth binding, is now on sale at the Office of this Journal, price, 7s. 6d. Cases for binding the Numbers contained in this Volume 1s. 6d. Index to Vol. XXVIII., 2½d., post free.**

**The Monthly Part for February is now ready. Price 6d., by post 8d.**

### HOSPITAL SATURDAY FUND.

### LIST OF AWARDS, 1900.

| GENERAL HOSPITALS. | 1900. £ | s. | d. |
|---|---|---|---|
| Charing Cross | 267 | 4 | 0 |
| French (Shaftesbury Avenue, W.C.) | 115 | 17 | 0 |
| German (Dalston) | 170 | 12 | 0 |
| Great Northern Central (Holloway Road, N.) | 235 | 0 | 0 |
| Guy's (St. Thomas's Street, Borough, S.E.) | 409 | 13 | 0 |
| Hampstead Hospital (Parliament Hill Rd., N.W.) | 61 | 10 | 0 |
| Homœopathic (Great Ormond Street, W.C.) | 143 | 15 | 0 |
| Italian (Queen Square, W.C.) | 60 | 0 | 0 |
| King's College (Lincoln's Inn, W.C.) | 311 | 15 | 0 |
| London (Whitechapel Road, E.) | 899 | 13 | 0 |
| Metropolitan (Kingsland Road, N.E.) | 147 | 13 | 0 |
| Middlesex (Mortimer Street, W.) | 404 | 1 | 0 |
| Ditto (Convalescent Home) | 42 | 0 | 0 |
| Miller (Greenwich Road, S.E.) | 76 | 13 | 0 |
| Ditto (Supplementary Award) | *70 | 0 | 0 |
| North London or University College (Gower Street, W.C.) | 283 | 15 | 0 |
| North-West London (Kentish Town Rd., N.W.) | 120 | 14 | 0 |
| Poplar (303 East India Road, E.) | 127 | 1 | 0 |
| Queen's Jubilee (Earl's Court, S.W.) | 55 | 13 | 0 |
| Royal Free (Gray's Inn Road, W.C.) | 273 | 9 | 0 |
| St. George's (Hyde Park Corner, S.W.) | 358 | 2 | 0 |
| St. John's (Twickenham) | 10 | 0 | 0 |
| St. Mary's (Paddington, W.) | 397 | 8 | 0 |
| St. Thomas's (Albert Embankment, S.E.) | 183 | 2 | 0 |
| Seamen's (Greenwich, S.E.) | 298 | 0 | 0 |
| Temperance (Hampstead Road, N.W.) | 189 | 19 | 0 |
| Tottenham Hospital (The Green, Tottenham) | 97 | 18 | 0 |
| West Ham (Stratford, E.) | 124 | 0 | 0 |
| West London (Hammersmith Road, W.) | 259 | 19 | 0 |
| Westminster (Broad Sanctuary, S.W.) | 267 | 17 | 0 |
| **COTTAGE HOSPITALS.** | | | |
| Beckenham | 30 | 0 | 0 |
| Enfield (N.) | 25 | 0 | 0 |
| Sidcup (Kent) | 20 | 0 | 0 |
| Teddington and Hampton Wick | 10 | 0 | 0 |
| Willesden (Harlesden Road, N.W.) | 10 | 0 | 0 |
| Wimbledon | 30 | 0 | 0 |
| Wood Green (N.) | 10 | 0 | 0 |
| Woolwich and Plumstead (Shooters' Hill) | 30 | 0 | 0 |
| **SPECIAL HOSPITALS.** | | | |
| *Cancer.* | | | |
| Cancer (Brompton, S.W.) | 128 | 2 | 0 |
| *Chest.* | | | |
| Brompton (S.W.) | 409 | 13 | 0 |
| City of London (Victoria Park, E) | 245 | 1 | 0 |
| Ditto (Supplementary Award) | *237 | 0 | 9 |
| Infirmary for Consumption (Margaret St., W.) | 40 | 0 | 0 |
| Ditto (Supplementary Award) | *26 | 5 | 0 |
| North London (Mount Vernon, Hampstead; Out-patient Department, 41 Fitzroy Sq., W.) | 145 | 10 | 0 |
| Ditto (Supplementary Award) | *68 | 10 | 3 |
| Royal (City Road, E.C.) | 137 | 17 | 0 |
| Ditto (Supplementary Award) | *174 | 16 | 6 |
| *Children.* | | | |
| Alexandra, for Hip Disease (Queen Sq., W.C.) | 140 | 0 | 0 |
| Ditto (Supplementary Award) | *10 | 10 | 0 |
| Belgrave (77-79 Gloucester Street, S.W.) | 66 | 12 | 0 |
| Cheyne, for Incurables (Cheyne Walk, Chelsea, S.W.) | 81 | 2 | 0 |
| Children's Cottage Hospital (Cold Ash, Newbury) | 5 | 5 | 0 |
| Children's Home Hospital (Barnet) | 8 | 8 | 0 |
| East London (Glamis Road, Shadwell, E.) | 196 | 1 | 0 |
| Ditto Convalescent Home | 22 | 8 | 0 |
| Evelina (Southwark Bridge Road, S.E.) | 85 | 1 | 0 |
| Home and Infirmary for Sick Children (Lower Sydenham) | 74 | 10 | 0 |
| Ditto ditto Convalescent Home | 12 | 12 | 0 |
| Hospital for Sick Children (Great Ormond St., W.C.) | 303 | 17 | 0 |
| Home for Incurable Children (Maida Vale, W.) | 12 | 0 | 0 |
| Leyton, Walthamstow, and Wanstead (Walthamstow, E.) | 74 | 10 | 0 |
| North-Eastern (Goldsmith Row, Hackney Road, N.E.) | 101 | 4 | 0 |
| Paddington Green (W.) | 99 | 6 | 0 |
| Ditto Convalescent Home | 9 | 16 | 0 |
| St. Mary's (Plaistow) | 100 | 4 | 0 |
| Royal [for Women also] (Waterloo Bridge Road, S.E.) | 92 | 6 | 0 |
| Victoria (Chelsea, S.W.) | 111 | 18 | 0 |
| Ditto Convalescent Home | 29 | 8 | 0 |
| *Dental.* | | | |
| Dental of London (Leicester Square, W.C.) | 43 | 16 | 0 |
| Ditto (Supplementary Award) | **5 | 12 | 0 |
| Guy's (Supplementary Award) | *10 | 10 | 0 |
| *Epilepsy, &c.* | | | |
| Hospital for Epilepsy, Paralysis, and other Diseases of the Nervous System (Portland Terrace, Regent's Park) | 39 | 12 | 0 |
| National (Queen Square, W.C.) | 235 | 18 | 0 |
| West End, for Diseases of the Nervous System (73 Welbeck Street, W.) | 93 | 4 | 0 |
| *Fever.* | | | |
| London Fever (Liverpool Road, N.) | 25 | 0 | 0 |
| *Fistula.* | | | |
| Gordon (278 Vauxhall Bridge Road, S.W.) | — | | |
| St. Mark's (City Road, E.C.) | 47 | 12 | 0 |
| *Heart.* | | | |
| National Hospital for Diseases of the Heart (32 Soho Square, W.) | 57 | 11 | 0 |
| *Incurables.* | | | |
| Hospital of St. John and St. Elizabeth (St. John's Wood, N.W.) | 20 | 0 | 0 |

### Lying-in.

| | £ | s. | d. |
|---|---|---|---|
| British (Endell Street, Long Acre, W.C.) | 35 | 13 | 0 |
| City of London (102 City Road, E.C. | 25 | 0 | 0 |
| Clapham Maternity (41 Jeffreys Road, Clapham, S.W.) | 25 | 0 | 0 |
| East End Mothers' Home (396 Commercial Road, E.) | 38 | 14 | 0 |
| General (York Road, Lambeth, S.E.) | 40 | 0 | 0 |
| Queen Charlotte's (Marylebone Road, N.W.) | 91 | 8 | 0 |
| Maternity Charity (Howard's Road, Plaistow, E.) | 35 | 0 | 0 |

### Ophthalmic.

| | £ | s. | d. |
|---|---|---|---|
| Central London (238A Gray's Inn Road, W.C.) | 72 | 19 | 0 |
| Royal London (City Road, E.C.) | 193 | 10 | 0 |
| Royal South London (St. George's Circus, S.E.) | 92 | 14 | 0 |
| Royal Westminster (19 King William Street, Strand) | 78 | 1 | 0 |
| Western (155 Marylebone Road, W.) | 41 | 0 | 0 |

### Orthopædic.

| | £ | s. | d. |
|---|---|---|---|
| City (Hatton Garden, E.C.) | 62 | 10 | 0 |
| National (234 Great Portland Street, W.) | 77 | 16 | 0 |
| Royal (297 Oxford Street, W.) | 44 | 11 | 0 |

### Scrofula.

| | £ | s. | d. |
|---|---|---|---|
| Royal Sea Bathing (Margate) | 21 | 0 | 0 |

### Stone.

| | £ | s. | d. |
|---|---|---|---|
| St. Peter's (Henrietta Street, W.C.) | 40 | 2 | 0 |

### Skin.

| | £ | s. | d. |
|---|---|---|---|
| Hospital for Diseases of the Skin (52 Stamford Street, S.E.) | 30 | 0 | 0 |
| Ditto (Supplementary Award) | *65 | 12 | 6 |
| St. John's (Leicester Square) | 40 | 10 | 0 |
| Ditto (Supplementary Award) | *118 | 13 | 0 |
| Western | 10 | 0 | 0 |
| London (Supplementary Award) | *41 | 9 | 6 |

### Throat and Ear.

| | £ | s. | d. |
|---|---|---|---|
| Central London (Gray's Inn Road, W.C.) | — | | |
| Ditto (Supplementary Award) | *68 | 10 | 0 |
| London (204 Great Portland Street, W.) | — | | |
| Metropolitan (Grafton Street, Tottenham Court Road) | 41 | 3 | 0 |
| Ditto (Supplementary Award) | *39 | 15 | 0 |
| Royal Ear (Supplementary Award) | *12 | 0 | 0 |

### Women.

| | £ | s. | d. |
|---|---|---|---|
| Chelsea (Queen's Elm, Fulham Road, S.W.) | 82 | 15 | 0 |
| Ditto (Supplementary Award) | *6 | 0 | 0 |
| Grosvenor [for Children also] (Vincent Square, Westminster, S.W.) | 66 | 12 | 0 |
| Hospital for Women (Soho Square, W.) | 102 | 12 | 0 |
| Invalid Asylum (High St., Stoke Newington, N.) | 40 | 0 | 0 |
| New (144 Euston Road, N.W.) | 94 | 12 | 0 |
| Samaritan Free [for Children also] (Marylebone Road, W.) | 113 | 6 | 0 |

### DISPENSARIES.

| | £ | s. | d. |
|---|---|---|---|
| Brixton (Water Lane, S.W.) | 28 | 13 | 0 |
| Chelsea (41 Sloane Square, S.W.) | 38 | 17 | 0 |
| City (46 Watling Street, E.C.) | 22 | 3 | 0 |
| Clapham (42 Manor Street, Clapham, S.W.) | 10 | 0 | 0 |
| East Dulwich Provident (Landells Road, East Dulwich) | 11 | 0 | 0 |
| Eastern (Leman Street, E.) | 37 | 9 | 0 |
| Farringdon (17 Bartlett's Buildings, Holborn) | 25 | 0 | 0 |
| Finsbury (Brewer Street, Goswell Road, E.C.) | 47 | 5 | 0 |
| Forest Hill Provident (2 Perry Villas, Perry Vale, S.E.) | 8 | 0 | 0 |
| Holloway (Palmer Place, Holloway Road, N.) | 33 | 15 | 0 |
| Islington (303 Upper Street, N.) | 43 | 19 | 0 |
| Kensington (Church Street, Kensington, W.) | 37 | 14 | 0 |
| Kilburn, Maida Vale, and St. John's Wood, General (13 Kilburn Park Road) | 23 | 11 | 0 |
| London (27 Fournier Street, Spitalfields, E.) | 15 | 0 | 0 |
| Metropolitan (9 Fore Street, E.C.) | 41 | 3 | 0 |
| Portland Town Free (Henry Street, St. John's Wood, N.W.) | 12 | 0 | 0 |
| Public (59 Stanhope St., Clare Market, W.C.) | 19 | 12 | 0 |
| Queen Adelaide (Pollard Row, Bethnal Green, E.) | 34 | 18 | 0 |
| Royal General (25 Bartholomew Close, E.C.) | 24 | 0 | 0 |
| Royal South London (St. George's Circus, S.E.) | 27 | 10 | 0 |
| St. George's and St. James's (King Street, Regent Street, W.) | 38 | 17 | 0 |
| St. John's Wood and Portland Town Provident (Ordnance Road, N.W.) | 14 | 0 | 0 |
| St. Marylebone (77 Welbeck Street, W.) | 16 | 16 | 0 |
| St. Pancras and Northern (126 Euston Road, N.W.) | 15 | 0 | 0 |
| South Lambeth (Wilkinson Street, Clapham Road, S.W.) | 28 | 8 | 0 |
| Stamford Hill and Stoke Newington (High Street, Stoke Newington) | 53 | 15 | 0 |
| Tower Hamlets (White Horse St., Stepney, E.) | 25 | 0 | 0 |
| Ditto (Supplementary Award) | *10 | 10 | 0 |
| Walthamstow (Orford Road) | 15 | 0 | 0 |
| Western (Rochester Row, Westminster, S.W.) | 51 | 4 | 0 |
| Western General (Marylebone Road, W.) | 66 | 2 | 0 |
| Westminster General (9 Gerrard St., Soho, W.) | 43 | 1 | 0 |

### CONVALESCENT HOMES.

| | £ | s. | d. |
|---|---|---|---|
| Baldwin Brown (Herne Bay) | 10 | 0 | 0 |
| Chelsea, for Women (St. Leonard's) | 48 | 13 | 0 |
| Ditto (Supplementary Award) | *1 | 1 | 0 |
| Children's (Brentwood) | 4 | 0 | 0 |
| Friendly Societies' (Dover) | 63 | 3 | 0 |
| Homœopathic (Eastbourne) | 10 | 10 | 0 |
| Invalid Children's Nursing Home (Winifred House, Wray Crescent, Tollington Park, N.) | 10 | 0 | 0 |
| King's College (Hemel Hempstead) | 53 | 7 | 0 |
| Kitto, Mrs. (Reigate) | 20 | 0 | 0 |
| London Samaritan Society (Supplementary Award) | *30 | 12 | 0 |
| Mary Wardell (Children, Scarlet Fever) (Stanmore) | 40 | 0 | 0 |
| Metropolitan (Walton-on-Thames and Broadstairs) | 270 | 4 | 0 |
| Metropolitan (Bexhill-on-Sea) | 270 | 4 | 0 |
| Morley House (St. Margaret's, near Dover) | 288 | 8 | 0 |
| Ditto (Supplementary Award) | *47 | 3 | 0 |
| Mothers' (Supplementary Award) | *86 | 9 | 0 |
| Richmond (Supplementary Award) | *60 | 0 | 0 |
| Rustington (Supplementary Award) | *60 | 0 | 0 |
| St. Andrew's (Clewer, near Windsor) | 96 | 0 | 0 |
| St. Andrew's (Folkestone) | 163 | 2 | 0 |
| Ditto (Supplementary Award) | *30 | 14 | 6 |
| St. John's (Children) (Brighton) | 40 | 0 | 0 |
| St. Leonard's (Children) (near Hastings) | 105 | 6 | 0 |
| St. Mary's (Shortlands) | 3 | 3 | 0 |
| Scott Memorial (Supplementary Award) | *295 | 8 | 6 |

### INSTITUTIONS FOR GRATUITOUS NURSING OF SICK POOR IN THEIR OWN HOMES.

| | £ | s. | d. |
|---|---|---|---|
| Bloomsbury (23 Bloomsbury Square, W.C.) | 20 | 0 | 0 |
| Chelsea and Pimlico (22 Tite Street, Chelsea, S.W.) | 12 | 0 | 0 |
| East London (49 Philpot Street, Commercial Road, E.) | 80 | 0 | 0 |
| Hammersmith and Fulham (Carnforth Lodge, Hammersmith, W.) | 12 | 0 | 0 |
| Haggerston and Hoxton (105 Nichol Square, Hackney Road, E.) | 24 | 0 | 0 |
| Kensington (1 Bedford Gardens, Kensington, W.) | 14 | 0 | 0 |
| North London (413 Holloway Road, N.) | 15 | 0 | 0 |
| Paddington and Marylebone (4 Randolph Road, Maida Hill, W.) | 17 | 0 | 0 |
| South London (Marmion Road, Lavender Hill, S.E.) | 18 | 0 | 0 |
| Southwark, Newington, and Walworth (37 West Square, St. George's Road, S.E.) | 13 | 0 | 0 |
| St. Olave's (23 St. James's Road, Bermondsey) | 25 | 0 | 0 |
| Westminster Nursing Committee (27 Bessborough Gardens, S.W.) | 9 | 0 | 0 |
| Woolwich, Plumstead, and Charlton (6 Russell Place, Woolwich) | 9 | 0 | 0 |

### MISCELLANEOUS.

| | £ | s. | d. |
|---|---|---|---|
| Hospital Saturday Fund, Distribution Committee | 1,360 | 0 | 0 |
| Hospital Saturday Fund, Additional Grant for 1900 | 250 | 0 | 0 |

| | £ | s. | d. |
|---|---|---|---|
| Hospital Saturday Fund, Additional Grant for 1901 ... ... ... ... ... ... | 300 | 0 | 0 |
| Hospital Saturday Fund, Surgical Appliance Committee ... ... ... ... ... | 1,020 | 0 | 0 |
| Hospital Saturday Fund, Surgical Additional Grant for 1900 ... ... ... ... ... | 287 | 0 | 0 |
| Hospital Saturday Fund, Ambulance Committee | 50 | 0 | 0 |
| National Society for Employment of Epileptics (Colony, Chalfont St. Peter, Bucks) ... ... | 12 | 12 | 0 |
| Friedenheim (Upper Avenue Road, Swiss Cottage, N.W.) ... ... ... ... ... | 15 | 15 | 0 |
| Invalid Children's Aid Association (18 Buckingham Street, Strand, W.C.) ... ... | 20 | 0 | 0 |
| Lock Hospitals ... ... ... ... ... ... | 25 | 0 | 0 |
| Buckhurst Hill Medical Provident Home .. | 3 | 3 | 0 |
| Buckhurst Hill Village Hospital ... ... | 3 | 3 | 0 |
| Royal Mineral Water Hospital (Bath) ... ... | 21 | 0 | 0 |
| St John Ambulance Brigade, Metropolitan Corps (St. John's Gate, E.C.) ... ... ... | 10 | 0 | 0 |
| Surgical Aid Society (Salisbury Square, E.C.) | 35 | 0 | 0 |
| Metropolitan Provident Medical Association ... | 125 | 0 | 0 |
| Ditto (Supplementary Award) | *161 | 0 | 0 |

SUMMARY. 1900.

| No. | | No. | £ | s. | d. |
|---|---|---|---|---|---|
| 28 | General Hospitals ... ... ... ... | 28 | 6,392 | 3 | 0 |
| 8 | Cottage Hospitals ... ... ... ... | 8 | 165 | 0 | 0 |
| 60 | Special Hospitals ... ... ... ... | 57 | 4,931 | 12 | 0 |
| 31 | Dispensaries ... ... ... ... ... | 31 | 909 | 12 | 0 |
| 20 | Convalescent Homes ... ... ... ... | 17 | 1,496 | 0 | 0 |
| 26 | Miscellaneous (including Ambulance, Distribution and Surgical Appliance Committees, also Institutions for the Gratuitous Nursing of the Sick Poor in their own homes) ... ... | 26 | 3,805 | 13 | 0 |
| 173 | ... ... ... Total ... | 167 | 17,700 | 0 | 0 |

* These are Supplementary Awards made by the Distribution Committee during the year, and are not included in the total of £17,700.

### THE STUDENT OF MEDICINE.

### APPOINTMENTS.

**H. Allen**, M.R.C.S., L.R.C.P.Lond., appointed Medical Officer for the Crediton Union. **A. August Ayton**, M.B., Ch.B.Edin., appointed Resident Medical Officer to the Victoria Hospital, Burnley, *vice* **J. J. Crauford**, M.B., Ch.B.Dublin, resigned. **Charles Arthur Brigstocke**, M.R.C.S.Eng., L.S.A., appointed Certified Factory Surgeon for the Haverfordwest District, co. Pembroke. **Leslie Davies**, M.B., appointed Health Officer for the Shire of Birchip, Victoria. **T. Percy Legg**, M.B., F.R.C.S., appointed Assistant Surgeon, with charge of out-patients, to the Royal Free Hospital. **H. F. Main**, M.B., appointed Health Officer for the Shire of Glenlyon, Victoria. **William Arthur Marris**, M.D.Lond., M.R.C.S., L.R.C.P. appointed Senior House-Physician to the National Hospital for the Paralysed and Epileptic, Queen Square. **J. Malcolm Mason**, M.D., F.C.S., D.P.H.,Camb., appointed Chief Health Officer for the Colony of New Zealand. **Percy F. Money**, M.R.C.S.Eng., L.R.C.P.Edin., appointed Government Medical Officer and Vaccinator at Goodooga, New South Wales. **D. J. F. O'Donoghue**, L.R.C.P., L.R.C.S.Irel., appointed Medical Officer for the Coom Dispensary District of the Killarney Union. **Clive Riviere**, M.D.Lond., M.R.C.S., M.R.C.P., appointed Pathologist and Registrar to the East London Hospital for Children, Shadwell. **P. J. Rockett**, M.B., appointed Health Officer for the Shire of Maffra, Victoria. **Ernest F. Sall**, M.R.C.S.Eng., L.R.C.P.Lond., appointed Second Assistant Medical Officer to the Graylingwell Hospital (West Sussex County Asylum), Chichester.

